# Defining climate change acceptance in undergraduate science student populations: A Delphi study

**DOI:** 10.1371/journal.pone.0356374

**Published:** 2026-08-25

**Authors:** Jessica R. Duke, Emily A. Holt

**Affiliations:** Department of Biological Sciences, University of Northern Colorado, Greeley, Colorado, United States of America; Max Planck Institute for Solid State Research, GERMANY

## Abstract

Acceptance of climate change often promotes pro-environmental behavior and encourages greater support of climate change policy and mitigation strategies. Despite its important role, no standardized definition of climate change acceptance exists in the published literature, leading to significant variability in how it is measured by researchers. Additionally, climate change is included in many educational frameworks as a vital curricular topic for establishing scientific literacy; however, student acceptance is neglected as a key factor. This ambiguity makes it difficult to compare research findings from studies measuring acceptance and further complicates educational efforts aimed at fostering climate literacy. The goal of the present study was to establish a consensus definition of climate change acceptance for undergraduate science student populations using a mixed-methods Delphi methodology. Our process integrated data from three sources: a preliminary framework of 14 constructs derived from a scoping review of 523 articles, a pilot survey of undergraduate students, and four iterative rounds of interviews and surveys with 210 experts and stakeholders in biology, chemistry, and geosciences. After multiple rounds of data collection and revision, expert consensus and stability of responses resulted in two definitions for climate change acceptance: the **Anthropogenic Definition**, which focuses on climate change occurrence and human causality, and the **Anthropogenic + Mechanistic Definition**, which also incorporates mechanisms of temperature and greenhouse gases. This bipolarity of opinion amongst participants suggests that the appropriate definition for an undergraduate student may depend on educational context or academic level. These rigorously researched definitions provide needed context for existing research instruments, a clearer target for future instruments, and guidance for post-secondary science educators on what constitutes acceptance at different levels.

## 1. Introduction

### 1.1 What is Acceptance?

Acceptance of a phenomenon is an active, voluntary process that requires an individual to make sense of evidence with which they are presented [1,2]. This “evidence” may be empirical, personal observation, or trusted testimony [2,3]. Acceptance, however, steps beyond learned information into belief. In science, acceptance of scientific principles and theories is foundational to scientific progress and innovation. Science is not just many, isolated facts but is a matrix of complex concepts that are socially bound, where paradigm shifts spur scientific revolutions [4] and progress accumulates [5] yet are tethered by community acceptance. These evolutions in science entail a period of critique and final acceptance by the community (e.g., atomic number in chemistry, [6]; Darwinian evolution in biology, [7]; continental drift in geology, [8]). This critique is contingent upon evidence [9], and often a diversity of evidence is needed to gain acceptance [10]. While acceptance of scientific ideas is important to scientists and is foundational to the progress of science itself, acceptance is critical to everyone including scientists-in-training and non-scientists.

We know that any “animated construct” (*sensu* [11]), in which science is applied and relevant to one’s “consequential everyday life situation” (p. 509), has the potential to entangle with ideologies and become contentious and suffer from rejection [12]. Acceptance, however, looks different for different science constructs [13]. Within the life sciences, much of the existing literature to explore and clarify a definition of acceptance has centered on the topic of evolution [14–16]. Studies of other critically important socioscientific topics within and beyond life sciences, notably climate change, are disconnected across the literature and lack comprehensive evaluation of what defines its acceptance.

### 1.2 Climate change acceptance in the literature

Research on climate change acceptance has largely focused on the outcomes that result from acceptance. A meta-analysis by Hornsey et al. [17] defined several outcomes of climate change acceptance including policy support, pro-environmental behavior, and support of mitigation strategies, with other studies also reporting links to concern [18] and attention or importance [19,20]. Studies have reported direct links between an individual’s level of acceptance and these outcomes, with many studies indicating positive benefits (e.g., increased performance of pro-environmental behaviors) [21] associated with acceptance. Other studies have examined what factors might contribute to someone accepting climate change or its antecedents (*sensu* [17]). Research has reported knowledge [22], environmental values [23], and understanding the scientific consensus [24,25] as antecedents that support acceptance of climate change.

Although these relationships have been reported, many of these studies do not explicitly define what constitutes acceptance, outside its outcomes and antecedents. A deeper dive into how individual studies measure climate change acceptance unveils ambiguity and inconsistency. Most studies do not overtly outline their working definition of acceptance of climate change. One of the most notable tools to measure beliefs and attitudes towards climate change in the US (Global Warming’s Six Americas, [26,27], never defines climate change acceptance and it can only be implied from the resulting “audiences” that scale by belief in global warming, concern, and motivation. Additionally, van der Linden et al. [20] define “Belief in Climate Change” as the belief that climate change is occurring, yet “Belief in Human Causation” is categorized as separate but related to acceptance of climate change. In contrast, van Stekelenburg et al. [25] measured belief with a single statement that combined these two constructs (i.e., “Human activities are causing climate change”, p.3). Further, Guy et al. [22] used a published instrument that measured three constructs: acceptance that it is occurring, anthropogenic causation, and its negative consequences. These examples expose the flaw in viewing climate change acceptance as a unified concept, upon which the research community has agreed. On the contrary, a definition based on expert opinion and the literature is sorely needed.

A lack of consistent definitions of climate change acceptance in the literature can be problematic. If studies implicitly define acceptance differently, then their findings cannot be compared yet this limitation may be unclear to readers. Further, researchers may claim their study supports that acceptance of climate change is associated with greater policy support [28]; yet, is it simply enough for someone to accept that climate change is occurring or is acceptance of human causation also necessary? Researchers have treated climate change acceptance as a monolith that leads to multiple outcomes but no current evidence supports this claim. Measuring and understanding climate change acceptance is undeniably important, but the absence of a universal definition diminishes the utility of past work and potentially contributes to misinformation if acceptance has more than one definition.

### 1.3 Climate change acceptance and education

Education is another context where lacking a clear definition of acceptance can be undoubtedly detrimental. Climate change education can help address and amend common misconceptions [29,30], increase climate change awareness [31], help students link climate change to their everyday lives [32], and foster climate literacy and knowledge [33]. Recognizing that our youth will become our next generation of voters, US educators have prioritized climate change as an integral topic in K-12 [34] and higher education curricula (e.g., Vision and Change [35] in Biology; Community Framework for Geoscience Education Research [36] in Geosciences; ACS Guidelines for Bachelor Degree Programs [37] in Chemistry). However, the primary focus in US formal education is to increase knowledge, understanding, and awareness. Yet evidence from research on other socioscientific issues (i.e., evolution education) suggests that *understanding* and *acceptance* are tied together in unpredictable ways [38–40]. Moreover, teaching climate change without a clear understanding of students’ acceptance of climate change and the bounds of that acceptance can hinder efficacious learning.

Undergraduate students are a unique population that have been largely understudied in relation to their acceptance of climate change [Duke et al., Accepted, 41]. Studies show that most college students recognize that climate change is occurring [41–45]; however, they continue to hold alternative conceptions of climate change mechanisms and causes [46,47], impacts [43,48], and its spatial and temporal scales [45]. Much of this prior work focuses on university students’ knowledge of climate change with only cursory investigation into their acceptance of climate change. Related to knowledge, some studies suggest that an individual’s willingness to participate in pro-environmental or sustainable behaviors is tied to education level [49,50]. While knowledge is undeniably important, it is one of many antecedents that contribute to an individual’s belief in climate change [17] and support of environmental action [49]. Stevenson et al. [18] found that adolescents’ personal belief in climate change is the most important factor influencing their concern for it; however, these beliefs are strongly influenced by their social connections with family and friends. Other research has found that direct experience with climate change events is also a contributor to acceptance, along with increased perception of risk [41,51]. Ultimately a person’s belief in climate change is influenced by a variety of factors that warrant expanding our focus beyond accumulation of knowledge.

### 1.4 Importance of consensus definition

Numerous surveys have measured climate change acceptance; however, these surveys vary significantly. Prominent US polling organizations, Pew and Gallup, frequently present public perceptions of the severity of climate change and individuals’ overall concern for it [52,53] as a proxy for acceptance. Conversely, Hornsey et al. [17] define both severity and concern as outcomes of acceptance rather than part of the construct itself. The Global Warming’s Six Americas survey [27] examines the public’s belief in the occurrence of climate change and human causality, while also assessing factors such as perceived risk and policy support, further complicating the landscape of published metrics. These three surveys represent a fraction of available metrics (Duke et al., Accepted) all of which suffer from inconsistencies in how climate change acceptance is measured [54]. In research, defining the topic of study is foundational to study design [55]; however, no consensus definition currently exists in the published literature. Without a standardized definition of acceptance, findings across studies remain incomparable, making it nearly impossible to synthesize data or determine whether shifts in public opinion reflect a true change in belief or merely a change in measurement.

Educational frameworks suffer the same defining woes of climate change acceptance. Several of these frameworks focus mainly on students acquiring knowledge of climate change (i.e., ACS Guidelines for Bachelor Degree Programs [37] in Chemistry; NGSS [29] in K-12), while others only mention how climate change poses unique educational challenges (i.e., Vision and Change [35] in Biology; Community Framework for Geoscience Education Research [36] in Geosciences). In the recently updated Four Dimensional Ecological Framework (4DEE) for undergraduate ecology students, human causality of climate change was added to the framework; however, general or explicit acceptance of climate change remains absent [56]. These frameworks are intended to guide educators in building curricula; yet climate change acceptance is notably lacking from these educational supports. Today’s students represent our next generation of scientists who will be challenged to innovatively tackle climate change; yet, acceptance is a neglected piece of their climate literacy. Since acceptance of climate change is linked to important outcomes such as support for mitigation strategies and policy reform, understanding the beliefs of our science students is integral for future progress.

In sum, climate action is contingent upon climate literacy, and climate change acceptance is plainly a critical element. Although numerous educational frameworks call for climate literacy, all sidestep acceptance as a factor that may enforce or derail educational efforts focused solely on knowledge gains. Clearly defining acceptance for specific student populations may aid educators in establishing effective interventions that consider different levels of acceptance. Further the plethora of existing research surveys touting to measure acceptance all lack a unifying definition. Therefore, there is no construct (or set of constructs) around which to establish validity [57], thus minimizing their comparability.

To fill the gap in research, the current study aims to rigorously develop such a definition of climate change acceptance. Specifically, our definition targets university science students as the focal population. For this study, science students are defined as those majoring in biology, chemistry, or earth and atmospheric science. These students represent a cohort regularly targeted for climate change knowledge assessment, yet their acceptance has been infrequently explored. Further, these specific disciplines were selected because their respective educational frameworks include climate change as an integral topic. By using a mixed-methods Delphi methodology, this research synthesizes insights from undergraduate students and their educators to establish a rigorous, population-specific definition of climate change acceptance which is currently absent in the literature.

## 2. Methods

### 2.1 The Delphi Method

The Delphi Method is often used by researchers seeking consensus on concepts for which little, inconclusive, or divergent evidence exists [58, p.1]. While climate change acceptance is frequently measured across the literature, no consensus definition exists [54]. Researchers have used this method to achieve similar goals such as establishing core principles in evolutionary medicine [59], defining the concept of “big data” [60], and defining learning outcomes in education [61].

In Delphi methodology, a panel of experts and stakeholders is recruited for multiple rounds of data collection with early rounds consisting of open-ended questions. Subsequent rounds of questions are then iteratively revised based on experts’ responses in earlier rounds [62] and participants are asked to rank options to establish priorities [62]. The number of rounds of data collection is dependent on stability across rounds and/or reaching a level of consensus amongst experts, yet three rounds is generally accepted as the standard [62]. The acceptable threshold for consensus is inconsistent across the literature [63] with some studies focusing more on stability of expert responses across each round of data collection [64], others relying on unanimity or near perfect agreement for medical-focused research [65], while others state that “A Delphi is considered complete when there is a convergence of opinion or when a point of diminishing returns is reached,” [66, p. 980]. For the purpose of the present study, we will follow von der Gracht’s [67] recommendation to secure the majority of agreement on a definition among respondents and seek stability across rounds. We also sought to collect in-depth rationale for respondents’ definition choice, jointly with their ideas of the ideal definition which was used to determine participants’ stability of response.

Our data collection for this study included interviews and surveys with students and educators (Table 1). For this study, educators in higher education served as our “experts” who best know the knowledge and skills related to climate change that is expected of our undergraduate student population of interest. Prior to this study, we conducted a scoping review of the literature (briefly described below) to examine how climate change acceptance has been measured across published studies which indicated that acceptance was multifaceted and potentially multidimensional [54]. This scoping review allowed us to create a preliminary codebook of potential constructs that might be important to climate change acceptance; however, this initial construct list was based on instruments that targeted populations beyond undergraduate science students. Conducting educator interviews as our first data collection step allowed us to ask experts detailed questions about their perceptions of undergraduate science students’ acceptance of climate change. These data allowed us to better understand our target population for our definition and how undergraduate science students’ acceptance of climate change may differ from other populations and narrow our questions for additional rounds of expert surveys.

**Table 1:** Overview of each Delphi round including its purpose, response type, and number and type of participants.

	Purpose of Research Activity	Response Type	Number of definitions offered	Number/type Participants
Pilot Student Survey	Test interview protocol questions	Open response	None	15 undergraduate students
Round 1	Confirm constructs for undergraduate science students’ acceptance of climate change	Open-ended interview questions	None	10 educators
Round 2	Rate climate change acceptance definitions; explore dimensions of climate change acceptance	Multiple choice and open response	15	57 educators
Round 3	Rate climate change acceptance definitions; explore definition choice for undergraduates at different academic levels	Multiple choice, open response, and ranking	2	26 educators
Round 4	Rate climate change acceptance definitions	Multiple choice and open response	2	109 educators

### 2.2 Scoping review of the literature

Between October 2023 and February 2024 we conducted a scoping review of the literature on climate change acceptance as part of a larger study (Duke et al., Accepted). A central requirement for article inclusion in our study was that the article include an instrument or items that measured climate change acceptance. In total, we identified 523 relevant articles from which we extracted 3,089 items measuring some aspect of climate change acceptance, 2402 of which were unique items [54]. These items were then thematically analyzed to explore what constructs are measured in existing instruments. Based on this analysis, we identified 14 constructs associated with climate change acceptance which represent a preliminary content framework for the domain [54]. These constructs helped inform the construction of an interview protocol for educators in the fields of biology, chemistry, and earth and atmospheric science, along with serving as a preliminary codebook to analyze interview data [54].

### 2.3 Participants

Data collection for this study was conducted with permission from the Institutional Review Board where the investigators are affiliated (IRB no. 2307050230). Consent was secured from all participants before conducting interviews and collecting survey data. Our recruitment for this study began in April 2024 and ended in July 2025. We recruited undergraduate science students and educators for this study within the fields of biology, chemistry, earth and atmospheric science from the United States. Since this study focuses specifically on undergraduate science students, “experts” were defined based on pedagogical experience within the target disciplines rather than broader climate science research. Therefore, our primary selection criterion was not extensive research expertise in the global field of climate change but rather demonstrated experience teaching climate change topics to undergraduate science students. For this study, an educator was defined as someone who met at least one of the following criteria: 1) teaches climate change concepts to undergraduate science students, and/or 2) is an educator for our target undergraduate science student population. Additionally, to document potential expertise within the subject, we asked educators to identify whether a significant portion of their academic research focused on climate change; however, this expertise was not an exclusion criteria. Participants for our study were recruited using purposeful sampling based on the above criteria who were employed in the home country of the research team. Identifying information was also collected from the participants during our data collection; however, participant data was deidentified in our reporting of results.

To ensure the consistency of question interpretation of our interview protocol, we conducted a pilot test in April 2024. Fifteen undergraduate students in a majors biology course at the home institution of the researchers were recruited to examine the readability of our interview protocol questions and explore variation of student responses. These were the only undergraduate science students recruited for this study.

Recruitment of educators for first round interviews began in May 2024 in the fields of biology, chemistry, and earth and atmospheric science. We sent out an initial 12 invitations to personal contacts within the target disciplines. Of these 12 individuals, six agreed to participate in our first round interviews. To increase our sample size, we asked participants to recommend colleagues who fit our expert criteria. An additional 12 invitations were sent, and an additional four interviewees were recruited. Data saturation (i.e., no new information arose) was reached after interviewing 10 participants, therefore, no additional recruitment for Round 1 occurred [68]. Of our 10 participants, four were from chemistry and three in each of the fields of biology and earth and atmospheric science.

Recruitment of educators for our second round of data collection began in December 2024. We contacted all 10 educators who participated in Round 1 interviews and 9 agreed to participate in our second round of data collection. Participating educators were asked to recommend colleagues who fit our expert criteria which allowed us to invite an additional 24 educators to participate, 12 of whom agreed to participate. A recruitment message was posted across several relevant professional listservs (i.e., Climate literacy network, ECOLOG, CER, GEO-ED Research) to further increase our sample size. Through this recruitment we found interest from experts in other relevant fields such as environmental science and discipline-based education research. An additional 36 participants from these listservs were recruited for a total of 58 participants (29.3% biology, 15.5% chemistry, 6.9% earth and atmospheric science, 10.3% environmental science, and 32.7% other).

For our Round 3 surveys, recruitment began in February 2025. We contacted all 58 participants from Round 2 and 26 agreed to participate (30.8% = biology, 23.1% = chemistry, 15.4% earth and atmospheric/geoscience, 7.7% environmental science, 15.3% other, 7.7% not reported). For our Round 4 surveys, educators were recruited during a July 2025 professional science conference. In total, 111 participants (73.9% = chemistry, 17.1% = chemistry education, 1.8% = biology, 7.2% = other) completed the survey. After our fourth round of data collection, we reached a general consensus and stability of response from participants across all rounds, ending our data collection [67]. Additional demographic characteristics of participants, beyond their discipline, from each round can be found in S1 Table.

### 2.4 Data collection

#### 2.4.1. Interview protocol.

Our interview protocl was adapted from related studies on evolution acceptance [69] and informed by our scoping review of the literature (S1 File). Our goal was to collect student and educator ideas about what elements were required, precluded, or did not interfere with climate change acceptance. The interview protocol was reviewed and edits were suggested by five external experts from the fields of biology, chemistry, geoscience, and psychometrics.

#### 2.4.2. Pilot test.

Prior to conducting interviews with educators, we tested our interview protocol questions with a group of 15 ecology undergraduate students. As part of their regular class activities, students completed an online survey which included the interview protocol questions (S1 File). Our goal was to identify student ideas about these topics to triangulate with expert ideas. This survey was administered during class within their course LMS. No identifying data were recorded from this survey, no individual or summarized responses are reported here, and retrospective consent was approved within our IRB.

#### 2.4.3. Interviews with educators – Round 1.

We conducted two pilot interviews to ensure that interview questions were interpreted by our sample population of experts consistent with our research goals. Final revisions occurred prior to the start of formal interviews. Our final interview protocol consisted of five open-ended questions, four of which were analyzed as part of this study (S1 File). Our first round interviews were conducted to distill the concepts that participants felt were integral for undergraduate science students’ acceptance of climate change.

Interview participants completed an online pre-survey prior to participating in the interview that consisted of four of our five interview questions (S1 File). This pre-survey was implemented because of the complex nature of our interview questions which we hoped would foster deeper, reflexive responses [70] and provide additional time during interviews to focus on novel elements of their responses. Participants then completed a 45–60 minute virtual interview that was audio recorded. In the interview, participants were asked to elaborate on the answers they provided in the pre-survey while also having the opportunity to contribute additional answers. Upon completing the interview, interviewees were asked to complete a demographic survey to help describe our population (S1 File). Participants who completed both the pre-survey and interview received a $10 Amazon gift card as compensation. Before analyzing interview data, all interviews were transcribed using Microsoft Word’s audio-to-text transcription feature and hand-checked by our team.

#### 2.4.4. Surveys with educators – Rounds 2–4.

Our second round of data collection consisted of an online survey developed from the results from our interviews in round one (S2 File). This survey consisted of four parts containing multiple choice and open response questions (Table 1). Participants were presented with 15 possible definitions of climate change acceptance, devised by our team during our scoping review of the literature and informed by student pilot data and Round 1 interviews. Parts 1 and 2 asked participants to select their preferred definition for undergraduate science students’ acceptance of climate change, to explain their selection, and to suggest other constructs that they believe should be added to the definition. Part 3 asked participants to provide feedback about all 15 acceptance definitions. Part 3 was not analyzed for the present study but rather used to help our team consider revisions to the definitions and prompts for subsequent rounds. Finally, Part 4 asked a series of demographic questions to help us describe our population of this round.

Our third round of data collection consisted of an online survey based on the results from our survey in round two (S2 File). This survey consisted of four parts containing ranking, multiple choice, and open response questions (Table 1). Part 1 asked participants to provide strengths and weaknesses of two definitions of climate change acceptance, which reflected the top-selected definitions from the previous round. Part 2 asked participants to select their top definition of the two provided and provide feedback on the wording of the definition they selected. Again, educator participants were asked whether any constructs were missing that should be added to the definition. Part 3 described two potential undergraduate science students (i.e., a first year freshman and final semester senior) and asked participants to select an appropriate definition of climate change acceptance for each hypothetical student. Participants were then asked whether they chose the same or different definitions for the two students and explain why. Finally, Part 4 asked a series of demographic questions to help us describe this round’s sample population.

Our fourth round of data collection consisted of an online survey based on results from previous survey rounds (S2 File). This survey consisted of two parts containing multiple choice and open response questions (Table 1). Part 1 asked educator participants to select their top definition choice of the two provided, which were the same two definitions provided in Round 3. Part 2 asked participants to explain why they chose this definition (S2 File). Finally, Part 4 asked a series of demographic questions to help us describe our population for this final round.

### 2.5 Data analysis

Given the multiple rounds of data collection, our data analysis was an iterative process which we began in April 2024, although it is described linearly below.

#### 2.5.1. Pilot test.

Our undergraduate student data were reviewed to look for patterns in responses. First, we reviewed the distribution of responses from students about ideas required to or not required to accept climate change. These ideas were compared to data from the next step from experts to confirm general alignment. Second, our team examined responses to ensure participants were answering as we expected to assess question functioning for subsequent rounds. Analyses from this step were formative to guide and confirm subsequent steps, thus not detailed here.

#### 2.5.2. Thematic analysis of interviews.

We conducted a thematic analysis of our 10 participant interview responses to four of our five questions (S1 File, questions marked with “*”). Our initial codebook was developed deductively from our scoping review of the literature and included all 14 of the climate change acceptance constructs represented in the preliminary content framework [54]. JD began coding interviews independently as they were completed and updated the codebook as new themes arose inductively. EH reviewed the codebook and confirmed coding of interviews as they proceeded. We continued collecting interviews until data saturation was reached (i.e., no new themes arose; [71]). Upon completing the interviews, the research team met to discuss the coded interviews and rectify any discrepancies in coding.

#### 2.5.3. Frequency counts and thematic analysis of surveys.

Analysis of survey data from Rounds 2–4, occurred at the completion of each round. We conducted frequency counts of participants’ choice of acceptance definition (from 15 definitions in Round 2, and two definitions in Rounds 3 and 4) to determine consensus for each round. The data were further numerically summarized from other survey questions requesting wording feedback, preferred definitions for a hypothetical first-semester versus a final-semester undergraduate, and demographics. Next, JD thematically analyzed participants’ rationale for why they selected their top definition and/or rejected others for each round, and EH reviewed this coding and any disagreement was discussed. JD also thematically coded expert participant responses in Round 3 to summarize their ideas about the strengths and weaknesses of the final two definitions and why their definitions for the two hypothetical students differed or were the same. All codes from these thematic analyses arose inductively through reading participant open-response questions.

## 3. Results

### 3.1 Content validity

Our process of developing a definition for climate change acceptance for undergraduate science students began by establishing content validity of the domain. Specifically, we sought evidence to support that the components of our definition represent the overall domain of climate change acceptance for undergraduate science students [55,57]. This was a vital first step in establishing a consensus definition to ensure we were comprehensively covering the full scope of constructs within the domain of climate change acceptance [55].

We triangulated three sources of data to provide evidence of content validity for our consensus definition. Our data sources included the framework of the core constructs of climate change acceptance [54], student survey responses (i.e., responses from our Pilot Survey), and expert or educator responses (i.e., responses from Rounds 2–4). Duke and Holt [54] synthesized 14 constructs of climate change acceptance, which was sourced from published research instruments from the literature. Our student participants commonly mentioned four of these constructs (i.e., Basic Belief, Anthropogenic Cause, Temperature, and Greenhouse Gases; Table 2) in the Pilot Survey. While other ideas, sometimes aligned with Duke and Holt’s [54] constructs, were mentioned by students, they aligned less with expert ideas. From our interviews with educators, we noted the same four core ideas as being integral for an undergraduate science student’s acceptance of climate change. This general agreement across multiple data sources provides strong evidence for content validity for our climate change acceptance definition [57].

**Table 2:** Definition of constructs mentioned by student and expert participants.

Theme	Definition	Freq. in Student Responses	Freq. in Educator Responses
Basic Belief	Personal perception that climate change is occurring, happening, or real (does not apply to perception of others’ beliefs).	93%	100%
Anthropogenic Cause	Perception that human actions or behaviors result in changes in the climate (e.g., driving cars, power plants that release greenhouse gases).	60%	60%
Temperature	Perception that temperatures are warmer now than they were in the past, recent past temperatures are warmer than farther distant past temperatures, and temperature is expected to be warmer in the future than it is now.	80%	30%
Greenhouse Gases	Perception that GHG concentrations are higher now than they were in the past and GHGs will continue to increase.	53%	20%
Credibility of Evidence	Perception that the process of science, information yielded, or expert propagating that information is reliable, trustworthy, and/or qualified as an expert.	27%	10%
Scientific Consensus	The belief that the majority of the scientific community has no doubt that modern climate change (since 1800) is occurring.	0%	10%

### 3.2 Definition consensus

After compiling multiple sources of content validity evidence [72] for our definition of climate change acceptance by undergraduate science students, we used language adapted from items analyzed in Duke and Holt [54], student survey responses, and educator interview responses to formulate 15 tentative definitions that covered these four core concepts. Each round of educator surveys targeted identifying the optimal definition and achieving participant consensus on a definition. Of the 15 definitions presented to participants in our second round of data collection, 50.9% (*n* = 29) selected the following definition as their top choice: *Climate change is occurring and temperatures and greenhouse gases are increasing globally and humans are causing it.* The second most popular definition (i.e., *Climate change is occurring and humans are causing it*) was selected by 19.3% (*n* = 11) of participants. The remainder of the definitions were selected by less than 10% of participants in Round 2, thus were removed from future rounds of data collection.

Of the two definitions included in our third round of data collection, the definition that included all four constructs (*Climate change is occurring and temperatures and greenhouse gases are increasing globally and humans are causing it*) was selected as the top definition by most participants (*n* = 18; 69%). We then revised wording of this definition based on expert feedback resulting in a final definition: *Climate change is happening, average temperatures and greenhouse gases are increasing globally, and human behaviors are causing it.* Although a general level of consensus was reached following our third round of data collection, our participants’ rationale for their top definition choice lacked richness, with many saying they selected the above definition because “*it was the most comprehensive”* or they felt that all undergraduate science students should possess the knowledge included in the broadest definition we offered.

**Fig 1:**
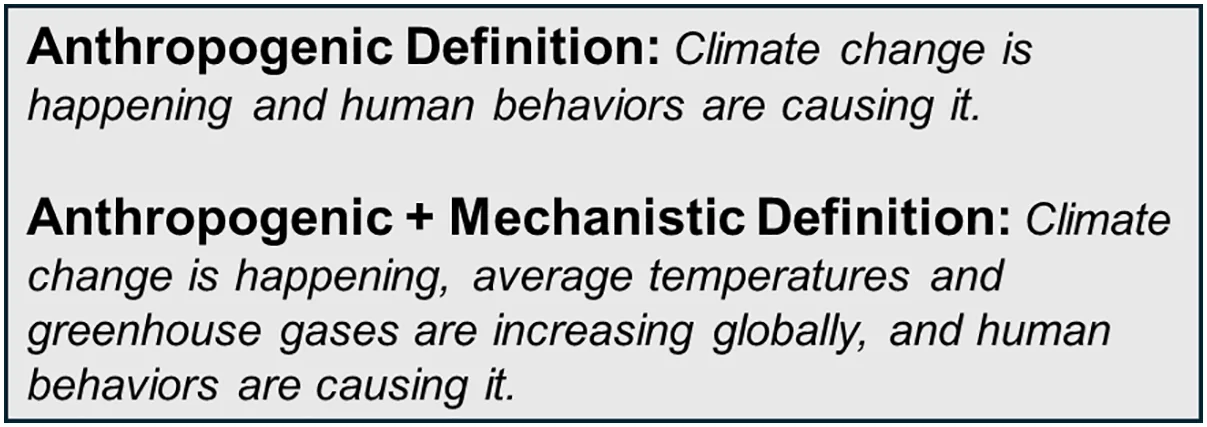
Consensus definitions of climate change acceptance.

Based on the lack of depth in participants’ rationale for definition choice in Round 3, we opted to collect additional survey data from additional educators. We included the two revised definitions in our fourth round of data collection. In line with recommended practice for Delphi methodology, our team communicated feedback about results from previous rounds to our Round 4 potential participants [73]. Specifically, our team presented percent agreement on all previous rounds, including that consensus (of nearly 70%) was reached on the more extensive definition, yet numerous participants, and our team, continued to, at least partially, disagree with this choice and that we were seeking opinions and more detailed explanations. In this final round our sample of educators preferred the simpler definition that included only two constructs (*Climate change is happening and human behaviors are causing it; n* = 70; 64%). Through four rounds of data collection, consensus oscillated between two definitions with strong support for each. von der Gracht [67] suggests that convergence and stability is the desired goal with the Delphi approach; therefore, we concluded that two definitions of climate change acceptance for undergraduate science students are relevant (Fig 1).

### 3.3 Rationale for definition choice

Beyond simply seeking consensus or stability on a definition for climate change acceptance for science undergraduate students (via percent agreement above a threshold), we aimed to describe the choices behind the definitions selected. These explanations became even more critical for our decision to retain both definitions as the final outcome. Further, participants’ explanations prompted further data collection and exploration in our methodology. Thematic coding of explanations allowed us to gauge richness in expert thinking and determine stability across rounds. By our fourth round of data collection, no new themes arose in our analysis of participant rationale for definition choice and our team agreed that we had reached thematic saturation for our qualitative responses [71]. Specifically, the stability of responses by experts manifested as bipolarity or equal division of opinions on climate change acceptance for undergraduate science students [74]. Upon reaching stability in our qualitative responses from experts along with establishing consensus of definition choice, we decided to cease data collection for our study [67]. When describing participant rationale for each survey round (Table 1) we will refer to our top two definitions as presented in Fig 1.

#### 3.3.1. More constructs are better…or not.

For our second round of data collection, the majority of participants selected the Anthropogenic + Mechanistic Definition as their top choice, citing its completeness as rationale for their selection (*n* = 7; 12%). One educator participant expressed, “*I chose this definition because it is the most comprehensive. Since students may have varying interpretations of what climate change means, providing a clear and precise definition is essential.*” While it was our goal to develop a comprehensive definition of climate change acceptance for undergraduate science students’, many of the educators did not elaborate on the importance of including all four constructs within the definition. To reduce cognitive load by our Round 2 participants, within the survey, the 15 definitions were ordered from one construct up to the final one with all four constructs. However, this may have led our participants to satisfice rather than optimize definitions [75]. We also noticed in Round 3 that our experts preferred the Anthropogenic + Mechanistic Definition, even with fewer choices and still overwhelmingly provided completeness as their primary rationale. To retrospectively explore whether participants might have favored definitions with even more constructs than the four outlined in the Anthropogenic + Mechanistic Definition, we reviewed participant responses about potentially “missing constructs” in the definitions provided as part of Round 2–4. Were educators simply choosing the Anthropogenic + Mechanistic Definition because it included the most measurable facets of acceptance or did researchers actually think all four constructs were integral to an undergraduate science student’s acceptance of climate change?

Round 2 data collection asked participants whether other constructs should be added to their selected definition (S2 File). Participants were first asked if the constructs Credibility of Evidence or Scientific Consensus should be included in a definition because these two specific constructs were mentioned by at least one expert during our Round 1 interviews (Table 2). When asked about Credibility of Evidence and Scientific Consensus, many participants (*n* = 25, 44% and *n* = 27, 47%, respectively) stated that although each construct is important neither should be included within a definition of climate change acceptance. A few participants felt that these constructs were unnecessary to acceptance and therefore should not be included in the definition (*n* = 6*,* 11% and *n* = 6, 11%, respectively). Although some participants felt that Credibility of Evidence and Scientific Consensus were necessary to the definition (*n* = 24, 41% and *n* = 21, 36%, respectively), overall the majority felt that neither construct should be added to the definition of acceptance (*n* = 31, 53% and *n* = 33, 57%, respectively). Additionally, participants were given an open response question which allowed them to indicate additional constructs they felt should be added to the definition of acceptance. The majority of participants either did not respond to the question (*n* = 28, 49%) or responded that no additions were needed (*n* = 8, 14%). In Rounds 3 and 4, we also asked participants if they would add or remove components from their chosen definition, and most stated they would make no changes to either definition. Some participants made suggestions to add components to the definition (e.g., consequences, solutions); however, the literature clarified these suggested constructs as external or peripheral to the domain of climate change acceptance [17,54]. Given that most of our participants in Rounds 2–4 did not suggest adding additional constructs to their chosen definition, we assume that the Anthropogenic + Mechanistic Definition was favored because participants agreed with the included four constructs and not simply because it had the greatest number of constructs. Further from these latter data, we suspect that definitions with more than our four constructs would not have been favored more highly than those presented.

#### 3.3.2. Knowledge and acceptance conflation.

Several educators who selected the Anthropogenic + Mechanistic Definition in Round 2 seemed to conflate “knowledge” with “acceptance” (*n* = 5; 9%). For example, one participant said they selected the Anthropogenic + Mechanistic Definition because, “*It is important for students to understand the relationship between greenhouse gases and increasing temperatures, humans’ role in greenhouse gas emissions, and the complexity of a changing climate.*” While knowledge is a documented antecedent of climate change acceptance, it is not part of the overall domain of acceptance [17,54].

Participants in Round 2 provided more concrete reasons for selecting the Anthropogenic Definition, with the majority stating they chose this definition because it contained all the key variables to measure undergraduate science students’ acceptance of climate change (*n* = 5; 9%; e.g., “*I chose this because as a lower level undergraduate, the key is to accept that the climate is changing (irrespective to increasing or decreasing temperatures) and that we are the ones doing [it] (irrespective of exactly how)”).* These participants recognized the importance of temperature and greenhouse gases for understanding climate change, but felt that they were unnecessary components of a students’ acceptance. Specifically, these participants separated the domains of acceptance and knowledge. Some participants also felt that the Anthropogenic Definition was more accurate (*n* = 3; 5%). For example, one participant’s rationale for not selecting the Anthropogenic + Mechanistic Definition was, “*Temperature change patterns are not consistent globally and are difficult to quantify. Greenhouse gasses are a cause, not the phenomenon itself.*” Additionally, a few educators (*n* = 2) felt that including temperature and greenhouse gases, as done in the Anthropogenic + Mechanistic Definition, might imply to students that these are the only important facets of climate change.

#### 3.3.3. Simplicity is a strength and weakness.

For our third round of data collection, we asked participants to describe the strengths and weaknesses of each definition prior to selecting which definition they preferred (S2 File) Participants reported that the main strength of the Anthropogenic Definition was that it was succinct (*n* = 15; 58%; e.g., “*It is very simple and straightforward. If someone responds in the negative, you can be sure that they understood the question and are disagreeing with it without reservation”)*. Beyond its simplicity, several (*n* = 7; 27%) participants felt that the Anthropogenic Definition contained the important aspects of climate change acceptance, *“It is concise and touches on the two controversial aspects of climate change. Is it happening and what is it being caused by?”*. In our fourth and final round of data collection, the majority of participants selected the Anthropogenic Definition (*n* = 70; 64%) with most choosing this definition for its simple and concise wording (*n* = 28). For example, one participant said “*I think with the two definitions, I prefer the simplistic version because when you start to introduce more constructs then there [are] more factors for a student to think about*”, seeming to reflect on the utility of these definitions in a classroom context. Other Round 4 participants (*n* = 22; 20%) selected the Anthropogenic over the Anthropogenic + Mechanistic Definition because they felt that climate change is more than just increased temperatures and greenhouse gases. One participant felt that the Anthropogenic + Mechanistic Definition has “*too many moving parts*” and felt that it “*limits climate change to just that* [temperature and greenhouse gases]*, neglecting other aspects of climate change*”. Another participant felt that including all four constructs within the Anthropogenic + Mechanistic Definition “*stray(s) people away from acceptance*” and again implies that *“greenhouse gases and temperature are the only reasons”* for climate change.

Alternatively, some participants (*n* = 6; 23% in Round 3) felt that the simplicity of the Anthropogenic Definition was a weakness, especially since the definition is intended for undergraduate science students (e.g., “*This definition is more in line with what I would expect for the baseline of an incoming middle/high school student. It is a little too simplistic for an incoming undergraduate student.”*). For many Round 4 participants that selected the Anthropogenic + Mechanistic Definition (*n* = 39; 36%), the majority chose this definition because they felt it more specifically spoke to what climate change is (*n* = 34; 31%) and expressed that it offered enough detail for students to understand what is meant by climate change (*n* = 8; 7%). For example, one participant stated that the Anthropogenic + Mechanistic Definition “*Gives specifics regarding climate change for students. The phrase climate change alone is too vague”*. Another participant felt that the Anthropogenic Definition “*doesn’t provide the definition of climate change”* while they liked how the Anthropogenic + Mechanistic Definition outlines “*specificity of the consequences of climate change and how it defines what climate change is*”. Ultimately the lack of detail in the Anthropogenic Definition was seen as a weakness for most educators, leading only eight (31%) to select this definition as their top choice in Round 3.

#### 3.3.4. The role of mechanisms in climate change acceptance.

Many Round 3 educators (*n* = 15; 58%) felt that the Anthropogenic Definition’s omission of the mechanisms of climate change was a significant weakness of the definition (e.g., *“For undergraduate science students who may not have much of a scientific background, simply saying climate change, without providing additional details for what that entails, could limit self-identification of climate change acceptance.”*). Further, these participants felt that not including the mechanism could leave the phenomenon of climate change open to too much interpretation, potentially leading to unreliable survey responses from students, e.g., “*There is very little detail about ‘climate change’ which is a complex group of impacts and there is room for debate and argument”.*

Even more Round 3 participants (*n* = 18; 69%) reported that the biggest strength of the Anthropogenic + Mechanistic Definition was its inclusion of the mechanisms of climate change (i.e., temperature and greenhouse gases). For example, one participant felt that including the mechanisms offered a direct way to link climate change to anthropogenic causality, *“This definition is better because it outlined the mechanism by which climate change occurs and how human activities are drivers of these mechanistic entities”*. The inclusion of mechanisms was thought to be the biggest strength of the Anthropogenic + Mechanistic Definition and the omission of mechanisms was deemed the greatest weakness of the Anthropogenic Definition, which probably explains why the majority of participants in Round 3 (*n* = 18; 69%) selected the Anthropogenic + Mechanistic Definition as their top choice. Although most participants preferred the Anthropogenic + Mechanistic Definition, some felt (*n* = 9; 35%) that the original wording of the definition was inaccurate (S2 File). A few participants offered suggestions for revising the definition, for example, “*It does not describe how humans are causing it; it needs a link between anthropogenic change and climate change”*. Our research team reflected on these suggestions during our revisions and several were incorporated to create our final wording (Fig 1).

#### 3.3.5. Learning progressions in climate change acceptance.

For the final part of our third round of data collection we asked participants to choose which definition is most appropriate for two hypothetical students (i.e., first-semester freshman and final-semester senior; S2 File). Of our 26 participants, 20 participants chose the same definition for both hypothetical students with the majority (*n* = 16; 62%) selecting the Anthropogenic + Mechanistic Definition as the best definition to capture undergraduate science students’ acceptance of climate change at both academic levels. Similar to Round 2, the majority of educators stated that the Anthropogenic + Mechanistic Definition contained elements that students should understand (e.g., *“These are basic concepts that any high-school level student should have learned”*), again, conflating knowledge/understanding of climate change and acceptance of it. Four participants selected the Anthropogenic Definition for both hypothetical students, with all four saying that this definition was the most baseline option for acceptance and that it should not change with education level. Finally, five participants from Round 3 selected the Anthropogenic Definition for the first-semester student and the Anthropogenic + Mechanistic Definition for a final-semester student. The majority of these participants cited knowledge progression as their main rationale for their selection of different definitions for each student.

## 4. Discussion

### 4.1 Importance of definition for climate change acceptance

Until now, no definition of climate change acceptance has been present in the literature. Despite this, a multitude of published instruments exist to measure this complex construct [Duke et al., Accepted, 54]. The American Education and Research Association’s (AERA) Standards for Educational and Psychological Testing [55] indicates that the validity of a research instrument and its results are contingent on having a clear and precise definition of the construct being measured. Developing instruments prior to establishing a definition of the construct can result in construct underrepresentation [76, p. 261]; therefore, existing, published instruments may not be measuring all integral aspects of acceptance. The two definitions identified during this study in conjunction with an established preliminary framework of climate change acceptance [54] provide a definition of the domain of climate change acceptance for undergraduate science students which will help researchers more accurately measure it.

For educators, these definitions indicate key constructs that experts believe are relevant to understanding undergraduate science students’ acceptance of climate change. Undergraduate educators aim to prepare their students to communicate the science of climate change effectively and make data-driven decisions when voting on policies that align with and aid in mitigating climate change. Understanding their acceptance may be a critical gateway for integrating climate change topics across college science curricula [77]; however, instructors must know what their students accept before they can develop and implement effective course curricula [78]. Our two definitions (Fig 1) provide educators with choices as to what aspects of acceptance they deem important for their courses and students. Instructors who wish to measure acceptance of climate change can use their preferred definition to select instruments for their student population; however, we caution researchers to critically evaluate the validity and reliability of the instrument before using it to draw conclusions of student acceptance or inform curricular development.

### 4.2 Definition Choice

Using a mixed-methods Delphi methodology we were able to significantly narrow the field of potential definitions of climate change acceptance for undergraduate science students to two definitions (Figure 1). Across four rounds of data collection, we reached consensus amongst experts (i.e., >50% agreement on at least one definition) on their selection of a definition [67]. Additionally, through the collection of rich qualitative data on experts rationale for definition choice we achieved stability or consensus of participant responses across each round of data collection [67,74]. Coupling expert percent agreement with their stability of responses increases the reliability [79] and trustworthiness [80] of our findings. In fact, Dajani et al. [74] posit that both stability and agreement are needed for true consensus and reporting one without the other may make conclusions meaningless.

In our study, experts consistently had a bipolarity of opinions on their definition selection. While we initially sought to identify one definition of climate change acceptance for undergraduate students, analysis of expert rationale made it clear that one definition may not be appropriate for all undergraduate science students and therefore, may be situationally dependent. Some of our experts cited learning progression as a potential reason for why two definitions may be appropriate depending on the academic level of the students being measured. Learning progressions describe how a students’ knowledge, understanding, and ability to use concepts evolves as they acquire new knowledge and progress in their educational career [81,82], and can be used as an evidence-based educational tool for mapping how students’ construct knowledge about scientific principles [83]. Our Anthropogenic Definition includes two constructs (i.e., Basic Belief and Anthropogenic Cause) and does not necessarily require students to possess prior knowledge to be able to assess these aspects of acceptance. In contrast, our Anthropogenic + Mechanistic Definition includes two additional constructs (i.e., Greenhouse Gases and Temperature) and implicitly requires students to possess prior knowledge of the mechanisms of climate change to assess this level of acceptance. Given this perspective, educators may find our Anthropogenic Definition to be useful for defining acceptance of climate change for freshmen or for students entering a new course where possessing prior knowledge of climate change is not expected. As students progress academically or by the conclusion of a course, educators may find our Anthropogenic + Mechanistic Definition to be more applicable as a target for acceptance for those students.

Choosing a specific definition for undergraduate science students is dependent on multiple factors. Similar to some of our experts, educators may prefer the Anthropogenic + Mechanistic Definition because it includes multiple aspects of climate change, all of which they may expect their students to agree with or understand as part of being a university science major. Our experts mentioned that these mechanisms were a requirement for student acceptance of climate change, and that targeting a definition without the mechanistic aspects may promote teaching for blind acceptance of climate change without understanding the science behind it. Perhaps the underlying concern of these experts is mirrored when novice students hold alternative conceptions about scientific ideas and processes; yet, these same students accept or claim to understand these scientific ideas. For example, students commonly hold misconceptions related to the physics concepts of force and motion [84], yet they may believe they understand its basic principles using flawed intuition and observation [85]. While these alternative conceptions may be counter to some specific learning goals for a course, they might not hinder acceptance of the phenomenon. In climate change education, alternative conceptions abound and can center on climate mechanisms [86].

Our Anthropogenic Definition strips away prior mechanistic knowledge from baseline acceptance of climate change, which may be more appealing for some instructors. Meanwhile others may feel both are required for “true acceptance”. Some research on climate change acceptance has reported that increasing mechanistic knowledge about climate change can lead to increases in its acceptance [30,87]; however, acceptance is complex and often mediated by other factors (e.g., political affiliation, geography) therefore these increases are not consistent across studies [88]. Some instructors may prefer using both definitions for different student populations. Ultimately, definition choice depends on instructors’ own course expectations, student learning goals, and the academic level of their students. The definitions presented here were developed through rigorous data collection with experts and stakeholders and grounded in the literature; therefore, instructors can be confident in either or both definitions as appropriate for undergraduate science students.

### 4.3 Limitations

While our panel possessed extensive pedagogical experience across target science disciplines, it was demographically homogenous (S1 Table) with the majority of experts identifying as white women. Because cultural, racial, and gender identity can shape how individuals perceive, teach, and discuss climate change, the consensus definition established here may reflect specific dominant perspectives within academia. Future work should deliberately center diverse racial, ethnic, and intersectional identities among both educators and students to ensure the construct of climate change acceptance is inclusive and representative of broader undergraduate populations. Additionally, the corpus of literature and human subjects data upon which the development of these definitions was based derived from literature of American populations and opinions of students and experts in the US. Therefore, our definitions are limited by this national scope, yet international expansions may provide obvious next steps for research. Finally, our study focused on undergraduate science students within the disciplines of biology, chemistry, and earth and atmospheric science, meaning these findings may not be generalizable to other science disciplines nor non-science populations.

### 4.4 Next steps

Our study outlines two potential definitions that instructors may choose to define climate change acceptance for undergraduate science students. While these definitions were built from ideas within university settings, they could be applicable for other populations (i.e., general public); although, additional data should be collected to determine appropriate adaptations. Additionally, the multitude of existing climate change acceptance instruments that exist in the literature were developed without a universal definition in place. As a parallel, Barnes et al. [89] owe inconsistencies in studies about evolution acceptance to a multitude of research instruments that were not guided by a reliable, consensus definition. In the climate change acceptance literature, the majority of instruments were developed specifically to measure climate change acceptance in the general public (Duke et al., Accepted) and therefore may not fully measure undergraduate student acceptance based on the definitions outlined in our study. The research and educational community could benefit from additional instruments that are modified or developed based on the definitions we outline in this manuscript.

## Supporting information

S1 Table Demographic characteristics of participants organized by round of data collection. *Notes*. *These data were collected as part of a formal presentation, therefore, demographic data collection was limited.
(DOCX)

S1 File Round 1 Delphi Materials. (DOCX)

S2 File Round 2–4 Delphi Materials. (DOCX)
