# Supplementary material for: Defining climate change acceptance in undergraduate science student populations: A Delphi study
Source: PLoS One. 2026 Aug 25;21(8):e0356374. doi: 10.1371/journal.pone.0356374 (PMC13505952; doi:10.1371/journal.pone.0356374)
Supplement: Supplementary file 1 [file pone.0356374.s001.docx]

**S1 Table: Demographic characteristics of participants organized by round of data collection.**

| **Demographic Characteristics** | ***Round 1 (n = 10)*** | ***Round 2 (n = 57)*** | ***Round 3 (n = 26)*** | ***Round 4 (n = 109)**** |
| --- | --- | --- | --- | --- |
| *Expertise as climate change researcher* | | | | |
| High | 2 | 12 | 3 | - |
| Moderate | 2 | 20 | 11 | - |
| Average | 3 | 16 | 9 | - |
| Low | 2 | 7 | 3 | - |
| None or did not respond | 1 | 3 | 1 | - |
| *Expertise as climate change educator* | | | | |
| High | 3 | 27 | 15 | - |
| Moderate | 4 | 13 | 5 | - |
| Average | 2 | 12 | 4 | - |
| Low | 0 | 5 | 2 | - |
| None or did not respond | 1 | 1 | 1 | - |
| *Discipline focus* | | | | |
| Biology | 3 | 17 | 8 | 2 |
| Chemistry | 4 | 9 | 6 | 82 |
| Earth and Atmospheric Science | 3 | 4 | 4 | - |
| Environmental Science | - | 6 | 2 | - |
| Other | - | 9 | 4 | 18 |
| Did not respond | - | - | 3 | 7 |
| *Level of courses taught* | | | | |
| Introductory undergraduate (100 or 200 level) | 8 | 40 | 20 | - |
| Advanced undergraduate (300 or 400 level) | 7 | 33 | 15 | - |
| Graduate | 4 | 48 | 22 | - |
| I have not taught in the past two years OR did not respond | 2 | 8 | 5 | - |
| *Position Type* |  |  |  |  |
| Postdoctoral Associate | 0 | 2 | 0 | 11 |
| Adjunct Faculty | 0 | 2 | 1 | 0 |
| Lecturer/Instructor | 0 | 4 | 2 | 4 |
| Assistant Professor | 3 | 15 | 10 | 17 |
| Associate Professor | 1 | 8 | 1 | 17 |
| Full Professor | 4 | 15 | 6 | 19 |
| Professional Scientist | 0 | 9 | 4 | 0 |
| Staff | 0 | 1 | 2 | 1 |
| Student | 0 | 3 | 1 | 33 |
| Retired professor | 1 | 2 | 1 | 0 |
| Prefer not to disclose | 1 | 4 | 0 | 1 |
| Gender Identity | | | | |
| Man | 5 | 20 | 12 | - |
| Woman | 4 | 36 | 15 | - |
| Non-binary / Non-gender conforming | 0 | 2 | 1 | - |
| Prefer not to disclose | 1 | 0 | 0 | - |
| *Race/ethnicity* | | | | |
| American Indian or Alaska Native | 0 | 0 | 0 | - |
| Asian | 0 | 0 | 0 | - |
| Black or African American | 0 | 0 | 0 | - |
| Hispanic or Latinx | 0 | 2 | 1 | - |
| Native Hawaiian / Pacific Islander | 0 | 0 | 0 | - |
| White | 9 | 53 | 27 | - |
| Other | 0 | 1 | 1 | - |
| Prefer not to disclose | 1 | 1 | 0 | - |
| *Notes*. *These data were collected as part of a formal presentation, therefore, demographic data collection was limited. | | | | |
