# Supplementary material for: Defining climate change acceptance in undergraduate science student populations: A Delphi study
Source: PLoS One. 2026 Aug 25;21(8):e0356374. doi: 10.1371/journal.pone.0356374 (PMC13505952; doi:10.1371/journal.pone.0356374)
Supplement: Supplementary file 2 [file pone.0356374.s002.docx]

**S2 File - Round 1 Delphi Materials**

*Expert Pre-Survey*

*Our survey also included an intro video (not provided here) explaining to experts the difference between belief, acceptance, and knowledge.*

The next four questions will ask you to reflect on acceptance of climate change in undergraduate science students.

Definition: When we refer to an “undergraduate science student”, we are including a college or university student who is enrolled in a science course (e.g. biology, chemistry, geosciences), and they do not necessarily have to major in a science discipline.

Q1: Tell us about any ideas or ways of thinking that you expect an undergraduate science student needs to adopt to fully accept climate change. Be as thorough as you can.

Q2: Tell us about any anti-science ideas or ways of thinking that you expect an undergraduate science student needs to reject to fully accept climate change. Please be as thorough as you can.

Q3: Tell us about any potentially relevant ideas or ways of thinking about climate change that you expect an undergraduate science student does not have to reject to fully accept climate change. Be as thorough as you can.

Q4: Our research goal is to develop a formal definition for acceptance of climate change.

Given your responses to other questions on this survey, what would be your idea for a starting definition for student acceptance of climate change?

*Expert Interview Protocol*

*Indicates questions that were analyzed for this study

Before revisiting the survey questions, I would like you to…

Q1: Take a moment to consider any one-on-one interactions that you may have had with an undergraduate science student where you discussed climate change (beyond asking about content). Have you had any of these conversations?

- **[If yes]** I invite you to share a story about your one-on-one interactions.
  - Why do you think that this student talked to you about this? Or about this topic?
  - What do you think were the goals of the student engaging in this interaction (questions, confirmation, complaints)?
- **[If no]** Our previous research suggests that faculty rarely have these one-on-one interactions with students about climate change.
  - Why do you think students don’t discuss climate change (beyond course content) with their science instructors?
  - How do you feel your relationship with your students might impact their interest or willingness to discuss climate change with you?

Thank you for those personal stories. Now let’s switch over to follow-up questions based on your responses to the pre-survey questions.

*Q2: Tell us about any ideas or ways of thinking that you expect an undergraduate science student needs to adopt to fully accept climate change. And some of your answers were XXX, YYY, and ZZZ. Tell me a bit more about XXXX. Having thought about these ideas more, do you have additional thoughts?

*Q3: Tell us about any anti-science ideas or ways of thinking that you expect an undergraduate science student needs to reject to fully accept climate change. And some of your answers were XXX, YYY, and ZZZ. Tell me a bit more about XXXX. Having thought about these ideas more, do you have additional thoughts?

*Q4: Tell us about any potentially relevant ideas or ways of thinking about climate change that you expect an undergraduate science student does not have to reject to fully accept climate change. And some of your answers were XXX, YYY, and ZZZ. Tell me a bit more about XXXX. Having thought about these ideas more, do you have additional thoughts?

* Q5: In the pre-survey you provided a starting definition for student acceptance of climate change [show participants definition]. After reflecting on your responses today, do you have any adjustments you might make to your original definition? If so, what might they be?

- We talked a lot about concepts and ideas that students need to adopt or reject to accept climate change. If we were to distill these ideas into a few critical pieces that are absolutely necessary for a student to accept climate change, what would these pieces be?
- These pieces may be layered for students at different levels (i.e., Freshman vs. senior), but what are the baseline concepts that every science student has to have to accept CC?

*Post Interview Demographic Survey*

1. How would you rate your expertise as a basic researcher in climate change science?
   a. High level of competence
   b. Moderate
   c. Average
   d. Low
   e. No level of competence
2. How would you rate your expertise as an educator about climate change?
   a. High level of competence
   b. Moderate
   c. Average
   d. Low
   e. No level of competence
3. In the last two years, what level of university courses have you taught? (select all that apply)
   a. Introductory undergraduate (100 or 200 level)
   b. Advanced undergraduate (300 or 400 level)
   c. Graduate
   d. I have not taught in the past two years
4. [What is your department/ science discipline] - open-ended
5. What is your position? (select all that apply)
   a. Postdoctoral Associate
   b. Adjunct Faculty
   c. Lecturer/Instructor
   d. Assistant Professor
   e. Associate Professor
   f. Full Professor
   g. Professional Scientist
   h. Self-identify (describe in the box)
   i. Prefer not to disclose
6. Which best describes your institution? (Select all that apply.)
   a. Doctoral University - very high research activity (R1)
   b. Doctoral University - high research activity (R2)
   c. Doctoral/Professional University
   d. Master’s College or University
   e. Baccalaureate College
   f. Associate’s College
   g. Historically Black University or College (HBCU)
   h. Hispanic Serving Institution (HSI)
   i. Tribal College or University
   j. Asian American and Pacific Islander Serving Institution (AAPISI)
   k. Other (describe in the box)
   l. Not associated with an institution of higher education
7. Which gender do you identify most closely with? (Select all that apply.)
   a. Man
   b. Woman
   c. Non-binary / Non-gender conforming
   d. Self-describe (describe in the box)
   e. Prefer not to disclose
8. What is your race/ethnicity? (Select all that apply.)
   a. American Indian or Alaska Native
   b. Asian
   c. Black or African American
   d. Hispanic or Latinx
   e. Native Hawaiian / Pacific Islander
   f. White
   g. Self-describe (describe in the box)
   h. Prefer not to disclose
9. What is the best email for us to send you an Amazon gift card as compensation for your participation? If you prefer not to receive one, just leave the box empty.
10. Would you be willing to be contacted by our team for participation in follow-up surveys or interviews?
    a. Yes
    b. No
    c. Maybe
11. We are actively recruiting expert participants for this study. Do you have any colleagues that you can recommend we contact as potential experts? If so, please list their name(s) and affiliation in the box below. - open response
