# Supplementary material for: Defining climate change acceptance in undergraduate science student populations: A Delphi study
Source: PLoS One. 2026 Aug 25;21(8):e0356374. doi: 10.1371/journal.pone.0356374 (PMC13505952; doi:10.1371/journal.pone.0356374)
Supplement: Supplementary file 3 [file pone.0356374.s003.docx]

**S3 File - Round 2-4 Delphi Materials**

ROUND 2 - EXPERT SURVEY

*Instructions:* The goal of this study is to develop a core definition of climate change acceptance in the context of undergraduate science students, so that it may be used to inform the development of survey measures of student climate change acceptance.

The survey contains multiple parts. **Parts 1** and **2** will focus on constructing and solidifying a core, "baseline" definition of climate change acceptance for undergraduate science students. You will be asked to first select a core definition that summarizes the basics of what it means to accept climate change, amend the language within your chosen definition, and then select additional statements that further clarify the core definition. We will use these results to assemble a complete definition; note that the definition components may be lightly edited for grammatical structure once they are assembled. **Part 3** of the survey will ask you to provide feedback on the multiple dimensions of climate change acceptance we have identified. **Part 4** asks about your background as a participant in this research study.

Please keep the goal of the study in mind as you answer the following questions. You can complete the current survey even if you did not participate in previous rounds of the study.

Important Definitions:

Undergraduate Science Student: a college or university student who is enrolled in a science course (e.g. biology, chemistry, geosciences) in the United States only. They do not necessarily have to major in a science discipline.

"Baseline" acceptance: refers to the level of acceptance of a first-year Freshman in college who has not yet received instruction on climate change at the university level.

Part 1: The following options offer different ways to phrase the core definition of what it means for an undergraduate science student to accept climate change. These statements were created based on data obtained from a scoping review of the literature and interviews with experts. Each of these options call for a different “baseline” level of what constitutes an undergraduate science student's acceptance of climate change. Note, there is redundancy in the below options by design.

P1, Q1: For the below options, please select your top choice of statement to be included in the baseline definition.

Climate change acceptance is agreeing that.....

1. climate change is occurring.
2. temperatures are increasing globally.
3. greenhouse gases are increasing globally.
4. humans are causing climate change.
5. climate change is occurring and temperatures are increasing globally.
6. climate change is occurring and greenhouse gases are increasing globally.
7. climate change is occurring and humans are causing it.
8. temperatures and greenhouse gases are increasing globally.
9. temperatures are increasing globally and humans are causing climate change.
10. greenhouse gases are increasing globally and humans are causing climate change.
11. climate change is occurring and temperatures and greenhouse gases are increasing globally.
12. climate change is occurring and temperatures are increasing globally and humans are causing it.
13. climate change is occurring and greenhouse gases are increasing globally and humans are causing it.
14. temperatures and greenhouse gases are increasing globally and humans are causing it.
15. climate change is occurring and temperatures and greenhouse gases are increasing globally and humans are causing it.

P1, Q2: Using the box below, please explain why you chose your top statement.

P1, Q3^1^: The below options, denoted in parentheses, will allow you to retain or adjust the wording of portions of the definition you previously selected as the best.

In some instances, new words or phrases have been given as options. If you believe these additions are not needed, please select * as the option within the drop-down menu(s).

Climate change acceptance is agreeing that...

- climate change is occurring.
  - climate change is (occurring/happening/real) (now/currently/unneeded clarification)
- temperatures are increasing globally.
  - (average/mean/unneeded clarification) (air/atmospheric/water/air and water/unneeded clarification) temperatures are (increasing/changing/rising/warming) (globally/worldwide/over large areas/locally) (at fast rate/ at an unprecedented rate/ at rate greater than can be explained by natural variation alone/unneeded clarification).
- greenhouse gases are increasing globally.
  - (greenhouse gases/carbon dioxide/greenhouse gas emissions) are/is increasing (increasing/changing/rising) (globally/worldwide/over large areas/locally).
- humans are causing climate change.
  - (human(s)/people/manmade/anthropogenic) (are/activity is/factors are/behaviors are/emissions are) (primary/largely/unneeded) (causing/ influencing/contributing to/ playing a role in/impacting/responsible for) (recent/current/unneeded clarification) climate change.

P1, Q4^2^: Your top choice did NOT include the following components: (climate change is occurring AND/OR temperatures are increasing globally AND/OR greenhouse gases are increasing globally AND/Or, humans are causing it)

Please explain why you did not include these elements within your top choice.

Part 2: Listed below are additional statements that could be added to the core definition. These additional statements may provide clarification (in measuring undergraduate science students' climate change acceptance) or might be unnecessary.

P2,Q1: Please review and provide feedback on the additional two statements and whether they are necessary for a baseline definition of climate change acceptance for a first year science undergraduate who has had no college science instruction.

Climate change acceptance is agreeing that.....

(1) Credible data exists to support that the climate is changing.

- Unnecessary, should not be added to the baseline acceptance definition
- Important, but should not be included in baseline definition
- Necessary, should be included in baseline definition
- Unsure

Climate change acceptance is agreeing that.....

(2) There is an overwhelming scientific consensus of climate change.

- Unnecessary, should not be added to the baseline acceptance definition
- Important, but should not be included in baseline definition
- Necessary, should be included in baseline definition
- Unsure

P2, Q2: Are there other elements not included in your top selected definition and listed above that you believe are necessary or important for baseline climate change acceptance in undergraduate science students? If so, please explain.

Part 3: This part asked experts to provide feedback on the multidimensionality of climate change acceptance, which we identified during our scoping review of the literature.^3^

Part 4: The following questions will ask you about your professional and personal background characteristics. These questions will only be used to help describe our study participants.

1. How would you rate your expertise as a basic researcher in climate change science?
   a. High level of competence
   b. Moderate
   c. Average
   d. Low
   e. No level of competence
2. How would you rate your expertise as an educator about climate change?
   a. High level of competence
   b. Moderate
   c. Average
   d. Low
   e. No level of competence
3. In the last two years, what level of university courses have you taught? (select all that apply)
   a. Introductory undergraduate (100 or 200 level)
   b. Advanced undergraduate (300 or 400 level)
   c. Graduate
   d. I have not taught in the past two years
4. [What is your department/ science discipline] - open-ended
5. What is your position? (select all that apply)
   a. Postdoctoral Associate
   b. Adjunct Faculty
   c. Lecturer/Instructor
   d. Assistant Professor
   e. Associate Professor
   f. Full Professor
   g. Professional Scientist
   h. Self-identify (describe in the box)
   i. Prefer not to disclose
6. Which gender do you identify most closely with? (Select all that apply.)
   a. Man
   b. Woman
   c. Non-binary / Non-gender conforming
   d. Self-describe (describe in the box)
   e. Prefer not to disclose
7. What is your race/ethnicity? (Select all that apply.)
   a. American Indian or Alaska Native
   b. Asian
   c. Black or African American
   d. Hispanic or Latinx
   e. Native Hawaiian / Pacific Islander
   f. White
   g. Self-describe (describe in the box)
   h. Prefer not to disclose
8. What is the best email for us to send you an Amazon gift card as compensation for your participation? If you prefer not to receive one, just leave the box empty.
9. Would you be willing to be contacted by our team for participation in follow-up surveys or interviews?
   a. Yes
   b. No
   c. Maybe
10. We are actively recruiting expert participants for this study. Do you have any colleagues that you can recommend we contact as potential experts? If so, please list their name(s) and affiliation in the box below. - open response

*Round 2 Notes:* ^1^The question that the expert received was dependent on their selection of a baseline definition in Part 1, Q1 using display logic. ^2^The statements experts received within parentheses were based on their response to Part 1, Q1 using display logic. ^3^The questions associated with this part of the survey are not provided as they are being analyzed as part of another project.

ROUND 3 - EXPERT SURVEY

*Instructions:* The goal of this study is to develop a baseline definition of climate change acceptance in the context of undergraduate science students, so that it may be used to inform the development of survey measures of student climate change acceptance.

This survey consists of three parts. In **Part 1** you will be shown each of the potential definitions and identify their strengths and weaknesses. **Part 2** will ask you to select your top choice of baseline definition. You will also be given the opportunity to provide feedback on the wording of your chosen definition. Finally, for **Part 3**, you will consider definitions of climate change acceptance and how they may relate to different undergraduate science student populations.

Please keep the goal of the study in mind as you answer the following questions. You can complete the current survey even if you did not participate in previous rounds of the study.

*Important Definitions:*

Undergraduate Science Student: a college or university student who is enrolled in a science course (e.g. biology, chemistry, geosciences) in the United States only. They do not necessarily have to major in a science discipline.

"Baseline" acceptance: refers to the level of acceptance of a first-year Freshman in college who has not yet received instruction on climate change at the university level.

Part 1: The following two definitions were selected from prior interviews and surveys with experts. We will consider each definition one at a time.

Definition 1: Climate change is occurring and humans are causing it.

Definition 2: Climate change is occurring, average temperatures and

greenhouse gases are increasing globally, and humans are causing it.

P1, Q1: Identify the weaknesses of the following definitions.

(1) Definition 1: Climate change is occurring and humans are causing it.

Please provide reasons or rationale for why "Definition 1" is NOT the best choice for representing undergraduate science students' acceptance of climate change (i.e., please argue against this definition).

(2) Definition 2: Climate change is occurring, average temperatures and greenhouse gases are increasing globally, and humans are causing it.

Please provide reasons or rationale for why "Definition 2" is NOT the best choice for representing undergraduate science students' acceptance of climate change (i.e., please argue against this definition).

P1, Q2: Identify the strengths of the following definitions.

(1) Definition 1: Climate change is occurring and humans are causing it.

Please provide reasons or rationale for why "Definition 1" IS the best choice for representing undergraduate science students' acceptance of climate change (i.e., please argue for this definition).

(2) Definition 2: Climate change is occurring, average temperatures and greenhouse gases are increasing globally, and humans are causing it.

Please provide reasons or rationale for why "Definition 2" IS the best choice for representing undergraduate science students' acceptance of climate change (i.e., please argue against this definition).

Part 2:

P2, Q1: In your opinion, please select your top choice for a baseline definition of an undergraduate science student's acceptance of climate change.

- Climate change is occurring and humans are causing it.
- Climate change is occurring, average temperatures and greenhouse gases are increasing globally, and humans are causing it.

P2, Q2^1^: The below options will allow you to retain or adjust the wording of portions of the definition you previously selected as the best. In some instances, new words or phrases have been given as options.

If you believe these additions are not needed, please select * as the option within the drop-down menu(s).

Climate change acceptance is agreeing that…

Please drag your favorite #1 option to the Blank 1 box and your favorite #2 option to the Blank 2 box. Remember, the * is for casing in which you feel the clarification for that blank is unnecessary.

Please drag your favorite #1 option to the Blank 1 box, your favorite #2 option to the Blank 2 box, your favorite #3 option to the Blank 3 box, and your favorite #4 option to the Blank 4 box. Remember, the * is for casing in which you feel the clarification for that blank is unnecessary.

Please drag your favorite #1 option to the Blank 1 box, your favorite #2 option to the Blank 2 box, and your favorite #3 option to the Blank 3 box. Remember, the * is for casing in which you feel the clarification for that blank is unnecessary.

Part 3: Consider two hypothetical student populations and which acceptance definition might fit best for them.

*Note. The following student profiles are strictly hypothetical. Names were randomly generated and bear no relation to actual students.

P3, Q1: Student 1: Loren is a first semester freshman in your Intro Chemistry course. While he took a variety of science courses in high school, this is his first experience taking a college science course.

(1) Which of the following definitions would best represent a baseline definition of acceptance of climate change for Loren?

- Climate change is occurring and humans are causing it.
- Climate change is occurring, average temperatures and greenhouse gases are increasing globally, and humans are causing it.

(2) Would you remove any components from your chosen definition? If so, please list these components and explain why you would remove them.

(3) Would you add any components to your chosen definition? If so, please list these components and explain why you would add them.

P3, Q2: Student 2: Tonya is a senior biology student in her final semester of college, having completed almost all of the required science courses to graduate. She is currently enrolled in an upper division biology seminar course, her final requirement for her degree.

(1) Which of the following definitions would best represent a baseline definition of acceptance of climate change for Loren?

- Climate change is occurring and humans are causing it.
- Climate change is occurring, average temperatures and greenhouse gases are increasing globally, and humans are causing it.

(2) Would you remove any components from your chosen definition? If so, please list these components and explain why you would remove them.

(3) Would you add any components to your chosen definition? If so, please list these components and explain why you would add them.

P3, Q3: If you chose the **same** baseline definition to define Loren and Tonya's acceptance of climate change, please explain why. If you chose **different** baseline definitions to define Loren and Tonya's acceptance of climate change, please explain why.

Part 4: The following questions will ask you about your professional and personal background characteristics. These questions will only be used to help describe our study participants.

The demographic questions presented here are the same as those described above in round 2, part 4.

*Round 3 Notes:* ^1^The responses provided to experts were based on their selection of a definition in Part 2, Q2 using display logic. Boxes as seen on the Qualtrics survey are not displayed here.

ROUND 4 - PROFESSIONALS SURVEY

**Question 1:** The following two definitions were selected from prior interviews and surveys with experts. Which do you feel is a better definition of undergraduate science students' acceptance of climate change?

Definition 1: *Climate change is happening, and human behaviors are causing it.*

Definition 2: *Climate change is happening, temperatures and greenhouse gases are increasing globally, and human behaviors are causing it.*

- I prefer definition 1
- I prefer definition 2

**Question 2:** RATIONALE: Please provide reasons or rationale for why your preferred definition is the best choice for representing undergraduate science students’ acceptance of climate change AND/OR reasons why the other definition is not as strong.

The following questions will ask you about your professional and personal background characteristics. These questions will only be used to help describe our participants.

1. [What is your department/ science discipline] - open-ended
2. What is your position? (select all that apply)
   a. Postdoctoral Associate
   b. Adjunct Faculty
   c. Lecturer/Instructor
   d. Assistant Professor
   e. Associate Professor
   f. Full Professor
   g. Professional Scientist
   h. Self-identify (describe in the box)
   i. Prefer not to disclose

In the below boxes, please include your name and email address if you are interested in helping us recruit students for our first field tests of the instrument Fall 2025 and/or Spring 2026. This does NOT commit you to help, rather simply expresses interest. Thank you!

Name: ________________________

Email Address: ___________________________
